# Risk factors and clinical outcomes of systemic cancer treatment delays in Filipino patients with solid tumor malignancy during the COVID‐19 pandemic: A single tertiary center study

**DOI:** 10.1002/cnr2.1426

**Published:** 2021-05-22

**Authors:** Jessa Gilda Pandy, Omar Maaño, Joanmarie C. Balolong‐Garcia, Jay T. Y. Datukan

**Affiliations:** ^1^ Section of Medical Oncology St. Luke's Medical Center Quezon City Philippines

**Keywords:** cancer, Covid‐19, Philippines, treatment delays

## Abstract

**Background:**

Cancer care during the Covid‐19 pandemic has been challenging especially in a developing country such as the Philippines. Oncologists were advised to prioritize chemotherapy based on the absolute benefit that the patient may receive, which outbalances the risks of Covid‐19 infection. The results of this study will allow re‐examination of how to approach cancer care during the pandemic and ultimately, help optimize treatment recommendations during this crisis.

**Aim:**

This study described the factors contributing to treatment delays during the pandemic and their impact on disease progression.

**Materials and results:**

This retrospective cohort study was done in St. Luke's Medical Center, a private tertiary healthcare institution based in Metro Manila, Philippines, composed of two facilities in Quezon City and Global City. Patients with solid malignancy with ongoing systemic cancer treatment prior to the peak of the pandemic were identified. Clinical characteristics and treatment data were compared between those with delayed and continued treatments. Multivariate analysis was done to determine factors for treatment delays and association of delays with disease progression and Covid‐19 infection. Of the 111 patients, 33% experienced treatment delays and 67% continued treatment during the pandemic. There was a higher percentage of patients on palliative intent who underwent treatment delay, and 64% of delays were due to logistic difficulties. Treatment delays were significantly associated with disease progression (*p* < .0001). There was no evidence of association between delay or continuation of treatment and risk of Covid‐19 infection.

**Conclusions:**

There was no difference in Covid‐19 infection between those who delayed and continued treatment during the pandemic; however, treatment delays were associated with a higher incidence of disease progression. Our findings suggest that the risks of cancer progression due to treatment delays exceed the risks of Covid‐19 infection in cancer patients implying that beneficial treatment should not be delayed as much as possible. Logistic hindrances were also identified as the most common cause of treatment delay among Filipino patients, suggesting that efforts should be focused into assistance programs that will mitigate these barriers to ensure continuity of cancer care services during the pandemic.

## 1 BACKGROUND

The Philippines has become one of the worst‐affected countries in Southeast Asia by the Covid‐19 pandemic, with the number of cases reaching over 600 000 in February 2021, and with at least 5000 new cases being reported daily.^1^ Delivering cancer care during the pandemic has been challenging, especially in a developing country, given the opposing risks of death from cancer versus death from severe Covid‐19 infection. Based on several retrospective studies, cancer patients who are receiving systemic anticancer treatment are generally thought to be at higher risk for severe Covid‐19 infection^2^, ^3^; hence, the American Society of Clinical Oncology (ASCO)^4^ and the European Society of Medical Oncology (ESMO)^5^ have developed modifications in cancer care guidelines in order to balance out the risks of Covid‐19 infection and benefits of prescribing chemotherapy and cancer treatment. The Philippine Society of Medical Oncology (PSMO) also recommended the prioritization of cancer treatment based on current cancer status and risk for Covid‐19 infection.^6^ Oncologists were advised to continue or delay treatment according to indication, timeline in the course of treatment, tolerance, and risk of cancer recurrence. Current recommendations include considering shorter treatment duration where feasible, delaying or postponing chemotherapy in patients in deep remission receiving maintenance therapy, shifting to oral systemic therapy, altering the chemotherapy schedule so that fewer hospital visits are needed.^4^, ^5^, ^6^

These recommended treatment delays, however, may lead to significant complications that may impact cancer outcomes such as disease progression, relapse, and mortality. It has been documented that delays in chemotherapy, in general, can have an adverse impact on survival.^7^ Several published studies have shown adverse outcomes in cancers such as breast, gastric, and colon with delaying adjuvant systemic treatment.^8^, ^9^ For patients with asymptomatic, incurable, metastatic cancer, palliative systemic treatment can be started immediately or can be delayed depending on factors such as disease nature, performance status, age, and co‐morbidities, and benefit from treatment.^10^ During the pandemic, additional factors that may contribute to treatment delays include travel inconvenience due to lockdown, financial issues, and accommodation issues. In the Philippines, a number of tertiary centers with cancer facilities have been designated as Covid‐19 referral centers, leading to temporary closures of chemotherapy centers.^11^

There is little data currently on how these pandemic‐related treatment delays and modifications affect cancer‐related outcomes. It also remains unclear whether treatment continuation increases the risk of Covid‐19 infection since the available data linking recent active oncologic therapy to Covid‐19 infection is mixed. In earlier studies^12^, ^13^ receipt of cancer therapy was associated with higher rates of Covid‐19 infection and worse outcomes. In contrast to previous data, a more recent study by Robilotti and colleagues from the Memorial Sloan Kettering Cancer Center showed that recent chemotherapy was not a determinant for severe Covid‐19 infection (OR 1.04, *p* = .845).^14^ This finding suggests that cancer treatments should not be delayed due to concerns about the virus.

It is important to characterize these pandemic‐related treatment delays to help cancer providers and healthcare systems develop effective strategies to manage cases during the current pandemic wave, subsequent waves, and future disasters. This study described the incidence of cancer treatment delays in cancer patients, the different factors that may have contributed to these delays and its impact on cancer‐related morbidity and mortality. This study also compared the rates of Covid‐19 infection among those who continued receiving treatment and those who did not. Recommendation on cancer care during a pandemic is still evolving and there is no “one size fits all” approach. The results of this study will allow re‐examination of how to approach cancer care during the pandemic and ultimately, help optimize treatment recommendations during this crisis.

## 2 METHODS

### 2.1 Study design and participants

This retrospective cohort study was carried out in two facilities, St. Luke's Medical Center Quezon City and Global City, Philippines. Cancer patients with histology proven solid tumor cancer of any stage with ongoing systemic cancer treatment prior to March 1, 2020, were included. The primary outcomes were rates of disease progression and Covid‐19 infection during the 6‐month period from March 1, 2020 to September 15, 2020, which represents the peak of the pandemic in the Philippines during that year. The included patients were divided into two groups: (1) those with continuation of treatment during the pandemic, and (2) those with treatment delays. Clinical retrospective data were retrieved from the medical records, including demographic features, clinical features, cancer histories, re‐evaluation images, and clinical outcomes. Reasons for treatment delay or modification were also recorded and were categorized into “financial,” “logistic,” or “treatment prioritization.” Patients with treatment discontinuations or delays due to chemotherapy‐related toxicities and/or disease progression while on systemic treatment were excluded. Two physicians (JP and OM) independently reviewed the data in duplicate, and any disagreements were resolved by consensus. The study was approved by the Ethics Review Committee of the St. Luke's Medical Center (No. SL‐20267).

### 2.2 Study definitions

Systemic cancer treatment includes cytotoxic chemotherapy, hormonal therapy, targeted therapy, and immunotherapy. Monotherapy includes treatment with only one type of agent, while combination therapy means treatment with more than one agent of systemic chemotherapy. Treatment delay was defined as discontinuation for more than 28 days or 4 weeks, due to pandemic‐related reasons, and not due to chemotherapy‐related toxicities or disease progression. A treatment delay of 4 weeks was shown in a meta‐analysis to be associated with worse survival across different cancer types.^15^ Treatment prioritization is the categorization of cancer patients into low, moderate, and high treatment priority based on the National Comprehensive Cancer Network Prioritization Guidelines for Covid‐19.^16^ “Logistic” reasons for treatment delays are those that result from the quarantine and lockdown protocols that took place during the pandemic and include reasons such as travel restrictions, suspension of public transportation, and closure of chemotherapy centers. “Financial” reasons for treatment delays are those that result from the inability of patients to procure cancer treatment drugs due to job disruptions and unemployment during the pandemic. Low income patients are defined as those with monthly income less than 21 914 pesos per month (about 500 USD), while mid to high income are patients with monthly income more than 21 914 pesos per month.^17^

### 2.3 Statistical analysis

Quantitative variables were presented as medians, and qualitative variables were presented by frequencies and percentages. Fisher's exact test and Pearson Chi test were applied to analyze the differences between groups according to demographics and clinical characteristics. Factors relating to treatment delay and their odds ratios (ORs) were analyzed by multivariable logistic regression. STATA software was used for statistical analysis. Fisher's exact test was done to investigate the effect of treatment delays on disease progression and Covid‐19 infection. The tests were all two‐sided with less than 5% type I error. The differences between groups were considered to be significant when the *p*‐value was less than .05.

## 3 RESULTS

### 3.1 Demographic and clinical characteristics

One hundred eleven (*n* = 111) cancer patients who underwent systemic treatment prior to the pandemic period between March 1, 2020, and September 15, 2020, were included. Of these, 33% experienced treatment discontinuation or delay, while 66.7% continued treatment during the pandemic. Majority were classified as mid to high‐income patients (94.59%) (Table 1).

**TABLE 1:** Demographic profile

	Patients (*n* = 111), %	Delayed treatment, *n* = 37 (33%)	Continued treatment, *n* = 74 (66.7%)	*p*‐value
Median age (Range), years	59 (21‐79)	58 (21‐78)	59 (24‐79)	.342
Sex				.891
Male	44 (39.64)	15 (40.54)	29 (39.19)	
Female	67 (60.36)	22 (59.46)	45 (60.81)	
Social service	6 (5.41)	5 (13.51)	1 (1.35)	.015
Private patient	105 (94.59)	32 (86.49)	73 (98.65)	

A significant proportion of patients had stage IV disease (68.47%) and there were more stage IV patients in the delayed treatment group (86.49% vs. 59.46%) (Table 2). There was no difference in type of treatment, and type of regimen between the two groups. The most common tumor types were breast, lung, and colon. Majority of patients with delayed treatment had lung cancer (40.54%), while most patients who continued treatment had breast cancer (29.73%). Overall, 78.38% received IV chemotherapy, 9.91% received targeted therapy, and 9.01% received immunotherapy. The most common indication for treatment was palliative followed by adjuvant. There was a significantly (*p* < .0001) higher percentage of patients on palliative intent who underwent treatment delay than continuation (86.49% vs. 48.65%), while patients on curative intent had less treatment delays (10.81% vs. 39.19%).

**TABLE 2:** Cancer characteristics and treatment profile

	Patients (*n* = 111), %	Delayed treatment, *n* = 37 (33%)	Continued treatment, *n* = 74 (66.7%)	*p*‐value
Tumor stage				.032
I	2 (1.80)	0 (0.00)	2 (2.70)	
II	13 (11.71)	2 (5.41)	11 (14.86)	
III	20 (18.02)	3 (8.11)	17 (22.97)	
IV	76 (68.47)	32 (86.49)	44 (59.46)	
Tumor type				.010
Lung	26 (23.42)	15 (40.54)	11 (14.86)	
Breast	31 (27.93)	9 (24.32)	22 (29.73)	
Genitourinary	4 (3.60)	1 (2.70)	3 (4.05)	
Gyne	10 (9.01)	6 (16.22)	4 (5.41)	
Gastric	5 (4.50)	2 (5.41)	3 (4.05)	
Lymphoma	3 (2.70)	0	3 (4.05)	
Head and Neck	8 (7.21)	0	8 (10.81)	
Colon	17 (15.32)	4 (10.81)	13 (17.57)	
Hepatobiliary	6 (5.41)	0	6 (8.11)	
Others	1 (0.90)	0	1 (1.35)	
Type of treatment				.939
Chemotherapy	87 (78.38)	29 (78.38)	58 (78.38)	
Hormonal therapy	3 (2.70)	1 (2.70)	2 (2.70)	
Immunotherapy	10 (9.01)	4 (10.81)	6 (8.11)	
Targeted therapy	11 (9.91)	3 (8.11)	8 (10.81)	
Type of Regimen				.0874
Monotherapy	26 (23.42)	9 (24.32)	17 (22.97)	
Combination	85 (76.58)	28 (75.68)	57 (77.03)	
Indication for treatment				<.0001
Neoadjuvant	10 (9.01)	1 (2.70)	9 (12.16)	
Adjuvant	33 (29.73)	4 (10.81)	29 (39.19)	
Palliative	68 (61.26)	32 (86.49)	36 (48.65)	

On multivariate analysis shown in Table 3, there was a significant decrease in treatment delay in patients with stage III disease (OR 0.24, 95% CI 0.066–0.90, *p* = .034) and breast cancer (OR 0.24, 95% CI 0.10–0.90, *p* = .032). There was no significant association with risk of treatment delay between other cancer types, type of treatment, type of regimen, and indication for treatment.

**TABLE 3:** Risk factors for Treatment Delay

	Odds ratio	95% CI	*p*‐value
Tumor stage			
I	1.0	—	—
II	0.25	0.052‐1.21	.084
III	0.24	0.066‐0.90	.034
IV	1.0	—	—
Tumor type			
Lung	1.0	—	—
Breast	0.24	0.10–0.90	.032
Colon	1.1	0.02‐2.68	.249
Gastric	0.49	0.25‐4.86	.900
Genitourinary	1.0	0.07‐3.43	.472
Gynecologic	1.0		
Head and Neck	0.26	0.06‐0.88	.32
Hepatobiliary	1.0		
Thyroid	1.0		
Type of treatment			
Chemotherapy	1.0	—	—
Hormonal therapy	1.0	0.87‐11.49	1.00
Immunotherapy	1.33	0.35‐5.10	.67
Targeted therapy	0.75	0.18‐3.04	.69
Type of Regimen			
Monotherapy	1.0	—	
Combination	1.08	0.43‐2.72	.874
Indication for treatment			
Neoadjuvant	—		
Adjuvant	1.24	0.12‐12.57	.86
Palliative	7.99	0.96‐66.65	.055

In those who had treatment delays, majority had reasons related to logistic difficulties (Table 4). Logistics‐related reasons, which included travel restrictions, lockdown policies, and closure of chemotherapy centers, were significantly associated with increased incidence of treatment delays (OR 35.36, 95% 11.74–106.47), *p* < .0001).

**TABLE 4:** Reasons for treatment delay

	Patients (%)	Treatment delay (OR)	95% CI	*p*‐value
Logistics	64	35.36	11.74–106.47	<.0001
Financial	8.5	1.0	—	
Recommendation‐based	34	2.37	0.88‐6.34	.086

A total of 39.64% of patients had disease progression (Table 5). There was a significant difference (<.0001) between rates of disease progression between patients who had treatment delays and continuation. Of the 37 patients with delayed treatment, 31 (83.78%) had disease progression. There was no significant difference (*p* = .239) in Covid‐19 infection rates between the two groups.

**TABLE 5:** Comparison of outcomes between those who experienced delays and those who did not

	Patients (%)	Delayed treatment, *n* = 37 (%)	Continued treatment, *n* = 74 (%)	*p*‐value
Disease progression	44 (39.64)	31 (83.78)	13 (17.57)	<.0001
Covid‐19 infection	15 (13.51)	7 (18.92)	8 (10.81)	.239

## 4 DISCUSSION

In this study, it was observed that there were more treatment continuations (66.7%) than delays (33%) in the included population. Similar to a study by Sun et al.^18^ on the impact of the outbreak on chemotherapy for cancer patients, they found that the rate of delay or regimen modification because of the outbreak was about 43.6%. In both studies, the percentages of treatment continuations were still higher than delays.

Another significant observation was that patients with early stage cancer were more likely to continue treatment, while patients with stage IV disease, on palliative treatment had higher rates of treatment delays. Based on the treatment prioritization guidelines proposed early on in the pandemic, patients with early‐stage disease on curative treatment were deemed urgent conditions and warranted continuation of treatment.^4^, ^5^ For many cancers, delays to treatment of 2–6 months will lead to a substantial proportion of patients with early‐stage tumors progressing from having curable to incurable disease.^19^ On the other hand, patients with cancers, which were advanced or metastatic, but who were stable or asymptomatic, were advised to consider delaying treatment.

In those who had treatment delays, about 40% were lung cancer patients. Lung cancer care during the pandemic has been more challenging than other tumor types since lung cancer patients are more prone to other nosocomial and community acquired infections. Early studies have also stated that lung cancer patients have a higher risk of getting infected with Covid‐19.^3^ These observations may have had an impact in treatment administration. Similar results have been shown in a recent study in Canada,^20^ which showed that 39.7% of lung cancer patients experienced delay in systemic treatment as a direct result of the pandemic. In another study by Fujita et al.,^21^ 9.1% of lung cancer patients requested to delay treatment due to fear of getting Covid‐19 infection.

We also observed that breast cancer patients were less likely to experience treatment delays compared to other types of cancer patients (OR 0.24, *p* = .032). In a survey done on breast cancer patients in the US^22^ showed that 32% reported experiencing delays in infusion therapies, while only 13% had delays in oral therapies. Based on treatment prioritization recommendations for breast cancer, the urgent conditions, which necessitate continuation of anticancer therapy include those for neoadjuvant, adjuvant, and first line metastatic treatment, which may explain a lower risk for treatment delays. In addition, a number of oral systemic options may be used in breast cancer treatment, hence, also making it easier to continue treatment.

**FIGURE 1:**
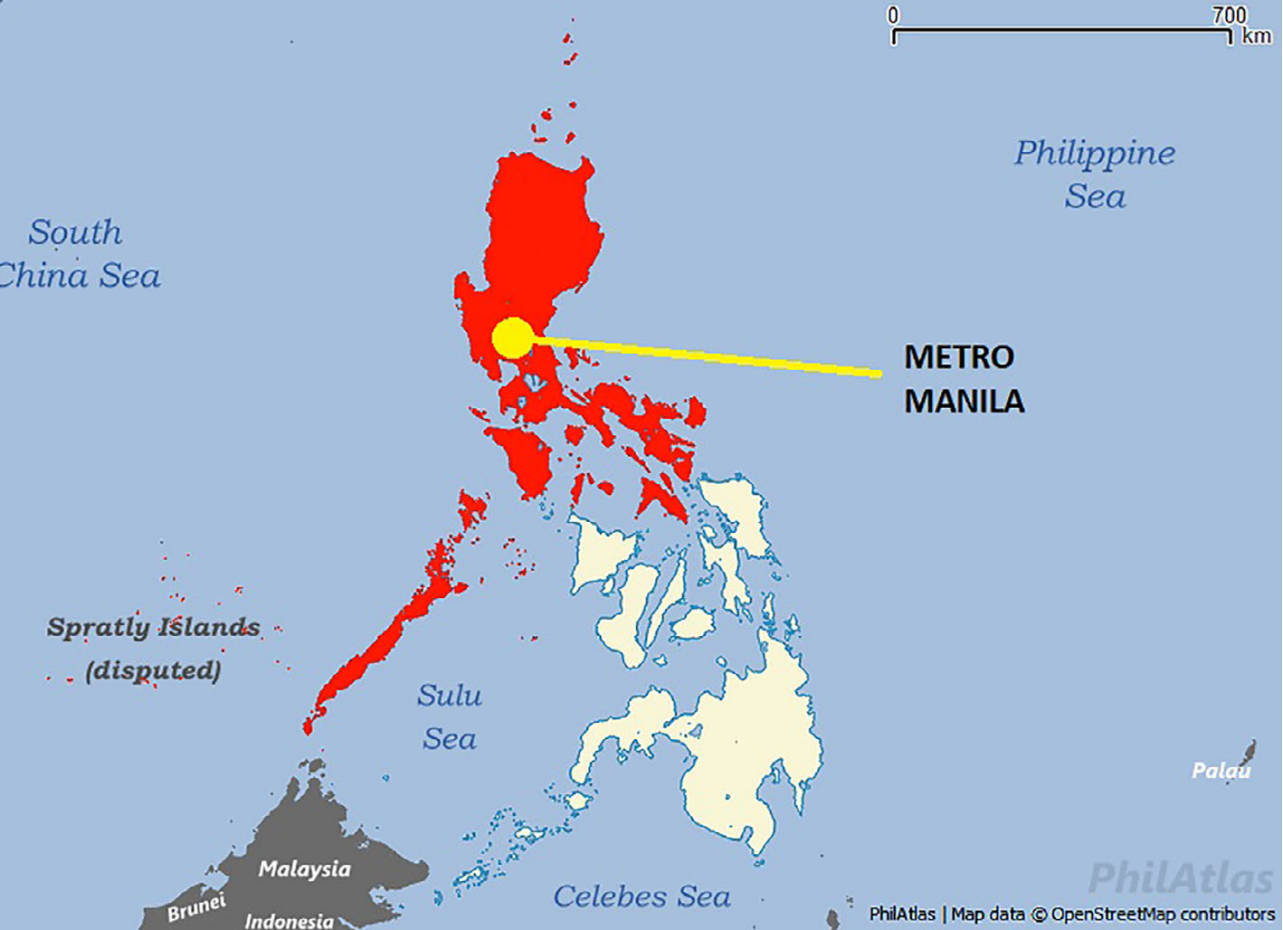
Map of the Philippines. (Areas in red) Luzon island placed on enhanced community quarantine. Modified from: https://filipinotimes.net/wp‐content/uploads/2020/03/luzon

Delays based on treatment prioritization occurred in 34% of the population, but were not shown to have a significant effect in the risk for treatment delays during the pandemic. This suggests that in our institution, delays are not solely based on the strategic approach of risk‐stratification, but rather on multiple factors. During the Covid‐19 pandemic, factors that might delay treatment can be grouped in two categories^7^: (1) patient‐related factors such as travel and financial issues, and (2) healthcare‐related factors such as shortage of manpower and hospital resources. In this study, the main reason for treatment delay was due to logistic reasons (64%, OR 35.36, *p*‐value <.0001). The entirety of the island of Luzon, including Metro Manila wherein our hospital is located, was first placed on enhanced community quarantine (ECQ) on March 16, 2020 (Figure 1).^11^ Under this period of lockdown, all modes of travel and movement had been restricted except for medical and essential services. Residents were not allowed to leave their homes except in the case of emergencies. The situation is further aggravated especially for patients who come from regions outside of Metro Manila since quarantine checkpoints have been placed in all borders. During this period, most cancer treatment facilities have been allocated to Covid‐19 patients, resulting in a large population of cancer patients who are not able to receive timely cancer treatment. At St. Luke's Medical Center, outpatient chemotherapy services were initially limited to only a few patients per day, resulting in delays in treatment scheduling. For a proportion of patients (8.5%), the pandemic has also exacerbated financial barriers to cancer treatment. The most common financial problems were reduced salary due to reduced work hours and unemployment. According to a recent report on the impact of Covid‐19 and national income, there was at least a 10% decline in income across the entire income distribution and a record‐high unemployment rate of 18%, meaning that about 7.3 million Filipinos had lost their jobs.^23^ Most of the included patients in this study were from the mid‐ to high‐ income sector, which may signify that they were also vulnerable to treatment delays due to financial losses.

An ESMO consensus on how to manage cancer patients during the pandemic was published last July 2020,^24^ advised oncologists not to discontinue or delay cancer treatment that may potentially impact overall survival. Although only 33% of patients had treatment delays, our findings support this ESMO statement that cancer treatment should not be delayed as much as possible. Of these patients, 83.78% experienced disease progression, which was a significantly greater proportion than in the treatment continuation group. In two modeling studies done in the UK^20^ and USA,^25^ the authors calculated over 6000 and 33 000 excess deaths, respectively, among cancer patients with cancer treatment delays. They estimated that due to pandemic‐related treatment delays, the number of excess deaths would peak in the next year or two.

The ESMO statement also discouraged classifying patients with cancer as highly vulnerable to Covid‐19 based on increasing evidence that many patients with solid tumors are not more vulnerable to Covid‐19 severe outcomes than the general population and that there is lack of convincing evidence that systemic therapy is associated with higher risk of Covid‐19 complications or mortality.^24^ In our study, there was no evidence that treatment delays led to a significant increase in Covid‐19 infection. Data from cohort studies such as CCC19^26^ and TERAVOLT^27^ commonly identified advanced age, male sex, poor PS, presence of comorbidities and active/progressing malignancy as risk factors for increased COVID‐19 mortality; on the other hand, cytotoxic chemotherapy, immunotherapy, and targeted therapies were not considered risk factors.

The limitations of our study include those that are inherent to the study's retrospective design. Data on reasons for treatment delays of the subjects were taken solely from physician's notes on the medical charts and are prone to information bias. We recommend to breakdown the logistic and financial reasons for delay into more specific reasons in order to describe further the possible problems that can be addressed during the pandemic. This study was performed in a tertiary hospital and the population used may not represent the general cancer patient population in the Philippines. Patients that were included were also limited to those who were able to follow‐up with evaluation studies and those who had complete medical records, hence, increasing the risk for selection bias. Our study was limited to patients with cancer delays and disease progression as the primary outcome. The impact of delayed cancer treatment varies across cancer types; since our population was heterogeneous with different types of cancers, we recommend studies focusing on specific types of cancers and how they are affected by treatment delays. We also recommend further studies on treatment modifications or regimen changes. Longer follow‐up can also be done to assess mortality and overall survival.

## 5 CONCLUSION

In conclusion, our findings suggest that the risks of cancer progression due to treatment delays exceed the risks of Covid‐19 infection in cancer patients, hence, implying that beneficial treatment should not be delayed as much as possible. This study also revealed a lack in adequate disaster preparedness that leaves cancer patients at high risk for treatment delay and poor outcomes. Oncologists, policy‐makers, and patients all have roles in moving toward optimal cancer care during this crisis. Cancer care during the pandemic is constantly changing and it is important that oncologists proceed with evidence‐based care. Current evidence on the impact of continuing or delaying cancer treatment during Covid‐19 times is still lacking in strength, and physicians should keep track of new studies and recommendation changes. Patients should also be assured that they can safely seek treatment, meaning that cancer treatment centers must take the necessary measures to protect the patients' safety by, at a minimum, having adequate supplies of personal protective equipment and sufficient access to rapid COVID testing. Lastly, the impact of logistic problems on treatment delays serves as a call for policy makers to develop interventions that will mitigate these logistic and financial barriers such as provision of transport services to increase accessibility to treatment centers, and allotment of adequate cancer treatment centers, which to ensure continuity of cancer care services during the pandemic. Despite the limitations of our study, our findings should help clinicians and policymakers in making evidence‐based decisions as we continue to ensure that our patients receive the best cancer care during this pandemic and future pandemics to come.

## CONFLICT OF INTEREST

The authors have stated explicitly that there are no conflicts of interest in connection with this article.

## AUTHORS' CONTRIBUTIONS

All authors had full access to the data in the study and take responsibility for the integrity of the data and the accuracy of the data analysis. *Conceptualization*, J.G.P., O.M., J.C.B.‐G., J.T.Y.D.; *Data Curation*, J.G.P., O.M.; *Investigation*, J.G.P.; *Formal Analysis*, J.G.P.; *Writing ‐ Original Draft*, J.G.P.; *Writing ‐ Review & Editing*, J.G.P., J.T.Y.D.; *Supervision*, J.T.Y.D.

## ETHICAL STATEMENT

The study was approved by the Ethics Review Committee of the St. Luke's Medical Center (No. SL‐20267). Patient consent was not required.

## Data Availability

All data generated or analyzed during this study are included in this published article and referenced articles are listed in the References section.
